# Correlations between disease-specific and generic health status questionnaires in patients with advanced COPD: a one-year observational study

**DOI:** 10.1186/1477-7525-10-98

**Published:** 2012-08-21

**Authors:** Sarah Wilke, Daisy JA Janssen, Emiel FM Wouters, Jos MGA Schols, Frits ME Franssen, Martijn A Spruit

**Affiliations:** 1 Program Development Centre, CIRO+, centre of expertise for chronic organ failure, Hornerheide 1, Horn, NM 6085, The Netherlands; 2 CAPHRI, Maastricht University, Maastricht, The Netherlands; 3 Proteion Thuis, Horn, The Netherlands; 4 Department of Respiratory Medicine, Maastricht University Medical Centre+ (MUMC+), Maastricht, The Netherlands; 5 Department of General Practice and Department of Health Services Research, Faculty of Health, Medicine and Life Sciences/CAPHRI, Maastricht University, Maastricht, The Netherlands

**Keywords:** Chronic obstructive pulmonary disease, Health-related quality of life, St. George’s Respiratory Questionnaire, Health status, Disease-specific health status, Generic health status

## Abstract

**Background:**

Longitudinal studies analyzing the correlations between disease-specific and generic health status questionnaires at different time points in patients with advanced COPD are lacking. The aim of this study was to determine whether and to what extent a disease-specific health status questionnaire (Saint George’s Respiratory Questionnaire, SGRQ) correlates with generic health status questionnaires (EuroQol-5-Dimensions, EQ-5D; Assessment of Quality of Life instrument, AQoL; Medical Outcomes Study 36-Item Short-Form Health Survey, SF-36) at four different time points in patients with advanced COPD; and to determine the correlation between the changes in these questionnaires during one-year follow-up.

**Methods:**

Demographic and clinical characteristics were assessed in 105 outpatients with advanced COPD at baseline. Disease-specific health status (SGRQ) and generic health status (EQ-5D, AQoL, SF-36) were assessed at baseline, four, eight, and 12 months. Correlations were determined between SGRQ and EQ-5D, AQoL, and SF-36 scores and changes in these scores. Agreement in direction of change was assessed.

**Results:**

Eighty-four patients (80%) completed one-year follow-up and were included for analysis. SGRQ total score and EQ-5D index score, AQoL total score and SF-36 Physical Component Summary measure (SF-36 PCS) score were moderately to strongly correlated. The correlation of the changes between the SGRQ total score and EQ-5D index score, AQoL total score, SF-36 PCS, and SF-36 Mental Component Summary measure (SF-36 MCS) score were weak or absent. The direction of changes in SGRQ total scores agreed slightly with the direction of changes in EQ-5D index score, AQoL total score, and SF-36 PCS score.

**Conclusions:**

At four, eight and 12 months after baseline, SGRQ total scores and EQ-5D index scores, AQoL total scores and SF-36 PCS scores were moderately to strongly correlated, while SGRQ total scores were weakly correlated with SF-36 MCS scores. The correlations between changes over time were weak or even absent. Disease-specific health status questionnaires and generic health status questionnaires should be used together to gain complete insight in health status and changes in health status over time in patients with advanced COPD.

## Background

Chronic obstructive pulmonary disease (COPD) is a highly prevalent chronic disease, characterized by expiratory airflow limitation, which is not fully reversible and usually progressive [1]. The Global initiative for chronic Obstructive Lung Disease (GOLD) classified the severity of COPD in mild (stage I), moderate (stage II), severe (stage III), or very severe (stage IV) according to the severity of airway obstruction and the presence of respiratory failure [1]. Despite optimal medical treatment, patients experience symptoms such as dyspnea, fatigue, and/or chronic cough [2]. Consequently, patients have a poor exercise capacity and limited performance in daily life activities [3,4].

Health status can be defined as “the impact of health on a person’s ability to perform and derive fulfillment from the activities of daily life” [5]. A patient’s self-reported health status thus includes health-related quality of life and functional status [5]. Patients suffering from COPD in all GOLD stages report an impaired health status, with worst scores reported by GOLD stage IV patients [6-8]. Therefore, improvement of health status is an important component in COPD management, including pulmonary rehabilitation [9-12].

A careful assessment of patient’s health status is necessary to identify targets for (non-) pharmacological interventions, as well as to evaluate the effects of these interventions [13]. Disease-specific health status questionnaires (i.e., related to a specific condition or group) or generic health status questionnaires (i.e., intended for general use, irrespective of the underlying disease) can be used as instruments to assess health status [14]. For instance, the Saint George’s Respiratory Questionnaire (SGRQ) is a commonly used instrument measuring disease-specific health status among patients with COPD [15-20], while the European Quality of Life-5 dimensions (EQ-5D) [18,19,21], Assessment of Quality of Life Instrument (AQoL) [22,23], and Medical Outcome Study 36-Item Short-Form Health Survey (SF-36) [15,17,24-26] are used to assess generic health status.

Previous cross-sectional studies found that disease-specific and generic health status questionnaires are moderately to strongly correlated in patients with COPD [17,19,27]. A longitudinal study among patients with newly detected COPD suggested that changes in a disease-specific health status questionnaire (Chronic Respiratory Disease Questionnaire, CRQ) are significantly correlated with the changes in several sections of a generic health status questionnaire (Nottingham Health Profile, NHP) [28]. However, longitudinal studies analyzing the correlations between disease-specific and generic questionnaires at different time points in patients with advanced COPD are lacking. Therefore, the aims of the current longitudinal observational study were: (1) to determine whether and to what extent a disease-specific questionnaire (SGRQ) correlates with generic health status questionnaires (EQ-5D, AQoL and SF-36) at four different time points in patients with advanced COPD; and (2) to determine the correlation between changes in these health status questionnaires during one-year follow-up.

## Methods

This study is an analysis of a longitudinal observational study concerning symptoms and care needs of patients with advanced chronic organ failure [29]. Methodological details of this study and cross-sectional data about health status, symptom distress, family caregiving and advance care planning in patients with advanced COPD were published before [29-34].

### Study population

Patients with advanced COPD were recruited by their chest physician at the outpatient clinic of one academic and two general hospitals in The Netherlands between January 2008 and June 2009. Patients were included if they had a physician-confirmed diagnosis of severe to very severe COPD (GOLD stages III and IV). Patients were excluded if they were not clinically stable for at least four weeks preceding enrolment (no hospital admission or major change in medication, according to the treating chest physician), if their pharmacological therapy was not optimal (according to the current available guidelines) and stable for at least two months preceding enrolment and if patients lived in a nursing home. All participating patients gave written informed consent. The Medical Ethical Committee of the Maastricht University Medical Centre+ (MUMC+), Maastricht, the Netherlands, approved this study (MEC 07-3-054). The study was registered at the Dutch Trial Register (NTR 1552).

### Instruments

Patients were visited by a member of the research team in their home environment. The following outcomes were assessed at baseline: demographics, weight and height, smoking status, current self-reported comorbidities (Charlson Comorbidity Index) [35], long-term oxygen therapy (LTOT), self-perceived mobility problems, dependency in personal care, and forced expiratory volume in the first second (FEV_1_). FEV_1_ was calculated from the flow-volume curve measured by a handheld pulmonary spirometer [36]. Severity of dyspnoea was measured using the modified Borg scale (range 0 [nothing at all] to 10 [maximal]) [37]. Symptoms of anxiety and depression were studied using the Hospital Anxiety and Depression scale (HADS), which is divided into an anxiety subscale (HADS-A) and a depression subscale (HADS-D) [38]. Total scores for each subscale range from 0 (optimal) to 21 points (worst). Patients were visited at baseline, and four, eight, and 12 months after baseline to assess disease-specific and generic health status using the questionnaires as described below.

### Disease-specific health status

Disease-specific health status was assessed using the SGRQ which consists of 76 items. The SGRQ provides three domain scores (symptoms, activity and impact) and a total score, ranging from 0 (optimal) to 100 points (worst) [39]. A recall period of three months was used.

### Generic health status

Generic health status was assessed using the self-administered questionnaires EQ-5D [21], AQoL [22] and the SF-36 [24].

EQ-5D is a five-item questionnaire consisting of several items: mobility, self-care, usual activity, pain/discomfort, and anxiety/depression [21]. Each item has three levels: no problems (1), some problems (2), and extreme problems (3). Higher scores on the several items indicate more severe problems. An index score is provided which ranges from −0.59 (worst) to 1.0 (best) [21]. In addition, patients rated their current health using a visual analogue scale (VAS). VAS score ranges from 0 (death or worst possible health) to 100 (best possible health).

AQoL consists of 15 items divided into five domains: illness, independent living, social relationships, physical senses, and psychological well-being [22]. Total score ranges from −0.04 (worst) to 1.00 (best) [40].

SF-36 consists of 36 items divided into eight domains: physical functioning, role-physical, bodily pain, general health, vitality, social functioning, role-emotional, and mental health. For each domain, scores range from 0 (worst) to 100 points (best) [24]. A Physical Component Summary measure (PCS) and a Mental Component Summary measure (MCS) are provided using norm-based methods with scores from a Dutch general population [41]. These summary measure scores are transformed to create a minimum and maximum possible score of 0 and 100 points. All scores below 50 points can be interpreted as below the general population norm [24].

### Statistics

Categorical variables were described as frequencies. Continuous variables were tested for normality and were described as mean and standard deviation (SD). Only patients who completed all four visits were included in the analyses. To compare baseline characteristics between patients who completed the study and those who withdrew from the study, an independent sample *t*-test or Mann–Whitney *U* test was used, as appropriate. For categorical variables, a Chi square test was used. Depending on the variable distribution, a paired sample *t*-test or Wilcoxon Signed-Rank test was performed to compare the baseline scores of the health status questionnaires with the follow up scores at four, eight, and 12 months. To visualize the correlations between the SGRQ total score and the EQ-5D index score, AQoL total score, and SF-36 PCS and MCS score at baseline, scatterplots were constructed. Furthermore, correlations of the changes in SGRQ total score and EQ-5D index score, AQoL total score, and SF-36 PCS and MCS score and the correlations of the changes in generic health status questionnaires between baseline and four months, four and eight months, and eight and 12 months were shown using scatterplots. To illustrate the correlations of the baseline EQ-5D, AQoL, SF-36 PCS and MCS scores and the correlations of the changes between these generic health status questionnaires, scatterplots were constructed. Additionally, bivariate correlations (Pearson’s product–moment correlation) were performed. Strength of correlation was classified as follows: absent (<±0.20), weak (±0.20 to ±0.34), moderate (±0.35 to ±0.50), and strong (> ± 0.50) [19,42]. The agreement in the direction of change in SGRQ total score and EQ-5D index score, AQoL total score, and SF-36 PCS and MCS score was determined. The four-months changes were classified into: no change, improvement and deterioration. For generic questionnaires, improvement is defined as a change >0 + (1.96*SE of mean change), deterioration is defined as a change <0-(1.96*SE of mean change), otherwise no change. For SGRQ improvement is defined as a change <0-(1.96*SE of mean change), deterioration is defined as a change >0 + (1.96*SE of mean change), otherwise no change. Cohen’s Kappa was used to test agreement. Kappa values were categorized as poor (<0.0), slight (0.00-0.20), fair (0.21-0.40), moderate (0.41-0.60), substantial (0.61-0.80), and almost perfect (0.81-1.00) agreement [43]. A p-value ≤0.05 was interpreted as statistically significant [44]. All statistical analyses were performed using SPSS for Windows, Version 19.0.

## Results

### Baseline characteristics

In total, 105 COPD patients were included (Table 1). Most patients had very severe COPD (GOLD stage IV: n = 77, 73.3%). Fifty-nine percent of the patients used long-term oxygen therapy. Patients were generally slightly overweight, and 85% of the patients experienced mobility problems. Patients who withdrew from the study were more frequently dependent in personal care than patients who completed the study.

**Table 1:** Baseline patient characteristics

	**Total sample (n = 105)**	**Study completed (n = 86)**	**Dropout (n = 19)**	**p-value**^**c**^
Male, n (%)	65 (61.9)	54 (62.8)	11 (57.9)	0.69
Age, years	66.3 (9.2)	65.7 (9.3)	68.8 (8.2)	0.18
BMI, kg/m^2 d^	26.3 (6.7)	26.5 (6.9)	25.5 (5.4)	0.99
Current smokers, n (%)	26 (24.8)	23 (26.7)	3 (15.8)	0.69
FEV_1_,% predicted^d^	34.1 (13.5)^a^	34.4 (13.6)^b^	32.7 (13.1)	0.60
Charlson index, points	2.5 (1.7)	2.4 (1.7)	2.7 (1.7)	0.50
Long-term oxygen therapy, n (%)	62 (59.0)	53 (61.6)	9 (47.4)	0.26
Borg scale (dyspnea), points	4.8 (2.0)	4.8 (2.0)	4.9 (2.1)	0.79
Borg scale (fatigue), points	4.6 (2.5)	4.4 (2.4)	5.3 (2.8)	0.15
HADS-A score, points	5.9 (4.5)	5.5 (4.2)	7.5 (5.6)	0.16
HADS-D score, points	6.3 (4.0)	6.0 (4.0)	7.7 (3.4)	0.10
Self-perceived mobility problems, n (%)	89 (84.8)	72 (83.8)	17 (89.5)	0.33
Dependent in personal care, n (%)	47 (44.8)	33 (38.4)	14 (73.6)	0.009

Values expressed as mean (standard deviation [SD]), number of patients (n), proportion (%).

*Abbreviations*: BMI, body mass index; FEV_1_, forced expiratory volume in the first second; HADS-A, Hospital Anxiety and Depression Scale, Anxiety subscale; HADS-D, Hospital Anxiety and Depression Scale, Depression subscale. ^a^ n = 102; ^b^ n = 83; ^c^ comparison between patients who completed the study and patients who withdrew from the study; ^d^ non parametric statistic tests were used because of skewed data.

### Health status

At baseline, patients generally had an impaired disease-specific and generic health status (Table 2). Patients who withdrew from the study reported worse scores on the SGRQ impact domain, several domains of the EQ-5D and AQoL, the EQ-5D index score and AQoL total score compared to patients who completed all four visits.

**Table 2:** Baseline health status

**Questionnaire**	**Domain**	**Total sample (n = 105)**	**Study completed (n = 86)**	**Dropout (n = 19)**	**p-value**^**c**^
EQ-5D	Mobility^d^	1.87 (0.39)	1.85 (0.39)	1.95 (0.40)	0.33
	Self-Care	1.70 (0.66)	**1.60 (0.62)**	**2.16 (0.69)**	**0.001**
	Usual activities	2.08 (0.65)	**1.99 (0.60)**	**2.47 (0.70)**	**0.01**
	Pain/discomfort	1.55 (0.66)	1.56 (0.64)	1.53 (0.77)	0.85
	Anxiety/depression	1.50 (0.67)	1.47 (0.66)	1.68 (0.67)	0.20
	Index score	0.51 (0.33)	**0.55 (0.30)**	**0.33 (0.37)**	**0.008**
	VAS	62.6 (14.0)	**64.1 (13.2)**	**55.8 (15.9)**	**0.02**
AQoL	Illness^d^	0.17 (0.17)	**0.18 (0.16)**	**0.12 (0.19)**	**0.04**
	Independent living	0.58 (0.30)	**0.63 (0.27)**	**0.36 (0.31)**	**<0.0005**
	Social relationships^d^	0.83 (0.20)	0.85 (0.20)	0.77 (0.20)	0.06
	Physical senses^d^	0.92 (0.11)	0.93 (0.09)	0.86 (0.18)	0.17
	Psychological wellbeing	0.87 (0.13)	0.97 (0.13)	0.85 (0.15)	0.50
	Total	0.46 (0.28)	**0.50 (0.27)**	**0.29 (0.30)**	**0.005**
SF-36	Physical Functioning^d^	21.0 (21.1)	22.3 (20.8)	15.5 (22.4)	0.07
	Role-physical^d^	37.1 (42.8)	38.4 (43.2)	31.6 (41.5)	0.61
	Bodily pain^d^	70.9 (29.5)	72.8 (28.3)	62.3 (34.0)	0.23
	General health	29.7 (19.1)	30.1 (19.2)	28.2 (19.3)	0.70
	Vitality	51.1 (18.9)	51.3 (19.1)	50.3 (18.7)	0.83
	Social functioning	65.0 (26.1)	67.0 (25.7)	55.9 (27.1)	0.10
	Role-emotional^d^	62.9 (44.9)	63.6 (44.7)	59.7 (46.6)	0.80
	Mental health	68.6 (19.9)	69.7 (19.3)	63.8 (22.2)	0.25
	PCS^d^	22.4 (9.6)	22.9 (9.2)	19.8 (11.3)	0.08
	MCS	47.3 (14.1)	47.7 (14.4)	45.1 (15.4)	0.48
SGRQ	Symptoms	58.6 (22.5)^a^	59.3 (22.9)^b^	55.4 (25.5)	0.52
	Activity^d^	74.8 (21.9)^a^	73.6 (21.3)^b^	80.1 (24.3)	0.06
	Impact	45.2 (18.5)^a^	**43.6 (17.7)**^**b**^	**52.7 (20.4)**	**0.04**
	Total^d^	56.5 (21.8)^a^	55.3 (16.0)^b^	61.5 (19.7)	0.09

Values expressed in points as mean (standard deviation [SD]). Bold printed numbers show significant (p ≤0.05) results. *Abbreviations*: EQ-5D, European Quality of Life-5 dimensions; VAS, Visual Analogue Scale; AQoL, Assessment of Quality of Life instrument; SF-36, Short Form 36-item health survey; PCS, Physical Component Summary measure; MCS, Mental Component Summary measure; SGRQ, St. George Respiratory Questionnaire. ^a^ n = 103; ^b^ n = 84; ^c^ comparison between patients who completed the study and patients who withdrew from the study; ^d^ non parametric statistic tests were used because of skewed data.

On average, patients who completed the study (n = 84) reported 1.1 (1.1) exacerbations during 12 months follow-up. Generic health status scores at baseline and 12 months after baseline were comparable between patients who had no exacerbations during 12 months (n = 33) and patients who had one or more exacerbations during 12 months (n = 51) (p >0.05). Patients who experienced one or more exacerbations during 12 months reported a worse SGRQ total score (58.3 (16.3) points) at baseline compared to patients who had no exacerbation during 12 months (50.7 (14.6) points), p = 0.03.

Table 3 shows the changes in health status over time. SGRQ total score, EQ-5D index score, and SF-36 PCS and MCS scores remained stable over time. Patients reported worse AQoL total scores at eight and 12 months compared to baseline.

**Table 3:** Health status at baseline, 4, 8 and 12 months

**Questionnaire**	**Domain**	**Baseline**	**4 months**	**8 months**	**12 months**
EQ-5D	Mobility^c^	1.85 (0.39)	1.83 (0.44)	1.90 (0.41)	**1.97 (0.32)**^**b**^
	Self-Care	1.60 (0.62)	1.67 (0.68)	**1.78 (0.66)**^**b**^	**1.76 (0.68)**^**b**^
	Usual activities	1.99 (0.60)	**2.15 (0.58)**^**b**^	**2.14 (0.58)**^**b**^	2.13 (0.70)
	Pain/discomfort	1.56 (0.64)	**1.40 (0.60)**^**b**^	1.55 (0.66)	1.47 (0.61)
	Anxiety/depression	1.47 (0.66)	**1.35 (0.63)**^**b**^	1.41 (0.62)	**1.33 (0.61)**^**b**^
	Index score	0.55 (0.30)	0.57 (0.31)	0.52 (0.32)	0.51 (0.31)
	VAS	64.1 (13.2)	61.7 (15.1)	61.9 (14.5)	**60.6 (13.4)**^**b**^
AQoL	Illness	0.18 (0.16)	**0.23 (0.16)**^**b**^	**0.24 (0.16)**^**b**^	**0.24 (0.17)**^**b**^
	Independent living	0.63 (0.27)	0.59 (0.28)	**0.54 (0.28)**^**b**^	**0.53 (0.29)**^**b**^
	Social relationships^c^	0.85 (0.20)	0.82 (0.21)	**0.81 (0.21)**	0.84 (0.18)
	Physical senses	0.93 (0.09)	0.94 (0.08)	0.93 (0.09)	0.94 (0.08)
	Psychological wellbeing	0.97 (0.13)	**0.90 (0.12)**^**b**^	0.90 (0.14)	**0.91 (0.11)**^**b**^
	Total	0.50 (0.27)	0.48 (0.26)	**0.44 (0.26)**^**b**^	**0.45 (0.24)**^**b**^
SF-36	Physical Functioning	22.3 (20.8)	19.9 (20.0)	21.6 (20.2)	20.3 (21.4)
	Role-physical^c^	38.4 (43.2)	37.8 (45.0)	48.8 (46.8)	41.9 (46.9)
	Bodily pain	72.8 (28.3)	75.2 (29.0)	72.0 (30.4)	76.5 (28.9)
	General health	30.1 (19.2)	27.6 (18.2)	28.5 (16.7)	28.2 (19.9)
	Vitality	51.3 (19.1)	53.9 (17.9)	53.4 (17.0)	52.7 (18.3)
	Social functioning	67.0 (25.7)	67.6 (26.7)	62.7 (28.6)	63.2 (28.7)
	Role-emotional^c^	63.6 (44.8)	67.8 (42.9)	67.1 (45.0)	70.2 (43.7)
	Mental health	69.7 (19.3)	70.6 (19.7)	69.4 (18.6)	69.4 (17.6)
	PCS	22.9 (9.2)	21.8 (9.6)	23.4 (10.5)	22.6 (10.4)
	MCS	47.7 (14.4)	49.5 (14.6)	47. 6 (14.4)	48.5 (13.2)
SGRQ^a^	Symptoms	59.3 (22.9)	56.4 (20.9)	57.2 (19.4)	58.2 (18.2)
	Activity	73.6 (21.3)	77.1 (18.2)	74.1 (22.1)	76.8 (19.7)
	Impact	43.6 (17.7)	43.6 (16.0)	42.3 (17.0)	43.3 (17.5)
	Total	55.3 (16.0)	55.9 (14.5)	54.3 (16.2)	56.3 (15.2)

Values expressed in points as mean (standard deviation [SD]). Bold printed numbers show significant (p ≤0.05) results. *Abbreviations*: please see legend Table 2. ^a^ n = 84; ^b^ p = ≤0.05 vs. baseline; ^c^ non parametric statistic tests were used because of skewed data.

### Correlations

Correlations between SGRQ total scores and the EQ-5D index score, AQOL total score, and SF-36 PCS and MCS scores were weak to strong at the four different time points (Table 4).

**Table 4:** Correlations between SGRQ total score and generic health status questionnaires

	**Baseline**	**4 months**	**8 months**	**12 months**
EQ-5D index score	−0.536	−0.439	−0.487	−0.421
AQoL total score	−0.617	−0.547	−0.587	−0.496
SF-36, PCS score	−0.679	−0.469	−0.464	−0.559
SF-36, MCS score	−0.489	−0.296	−0.314	−0.285

n = 84, all correlation: p ≤0.01.

*Abbreviations*: Please see legend Table 2.

**Figure 1:**
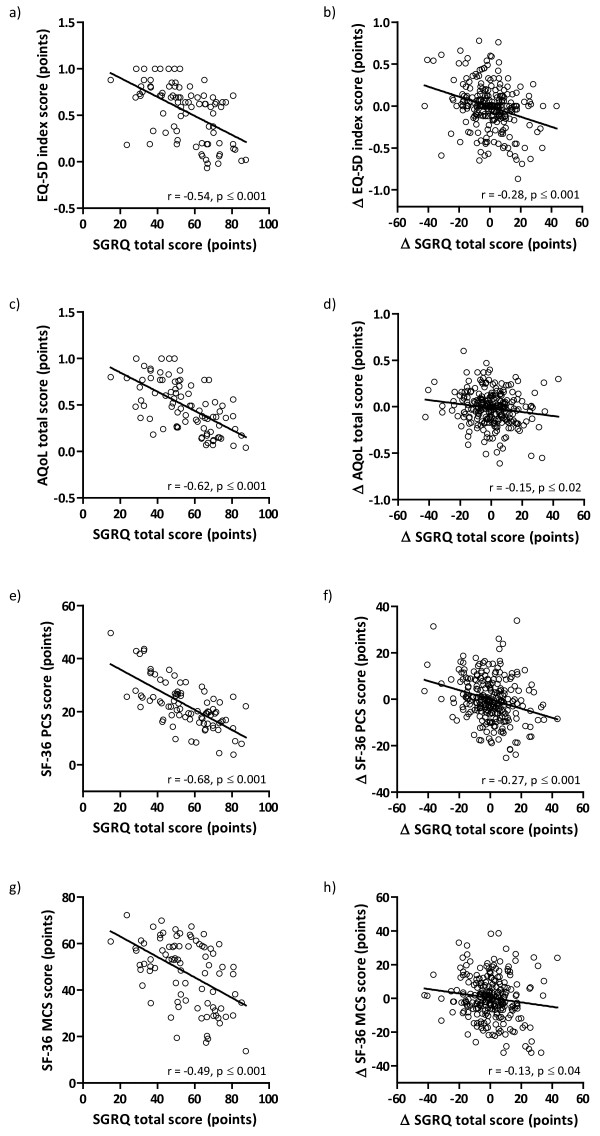
Correlation between SGRQ total score and EQ-5D index score (a), AQoL total score (c), SF-36 PCS score (e) and SF-36 MCS score (g) at baseline and correlation between changes between 0–4 months, 4–8 months and 8–12 months of SGRQ total score and changes of EQ-5D index score (b), AQoL total score (d), SF-36 PCS (f) and SF-36 MCS score (h) (all data points together); n = 84; Δ, change in.

Figure 1a to 1h show the correlations between the SGRQ total scores and EQ-5D index scores, AQoL total scores and SF-36 PCS and MCS scores at all time points, and the correlations between the changes in SGRQ total scores and the changes in EQ-5D index scores, AQoL total scores and SF-36 PCS and MCS scores. At baseline, disease-specific and generic health status questionnaires were moderately to strongly correlated, while the correlations between the changes were weak or absent.

**Figure 2:**
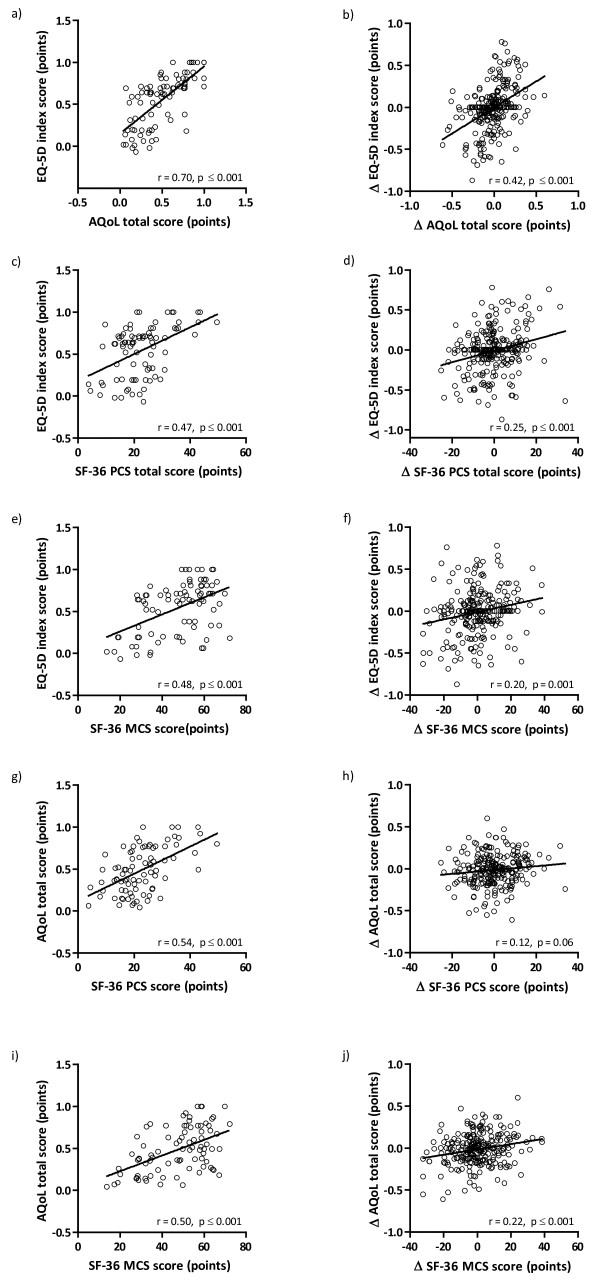
Correlation between EQ-5D index score and AQoL total score (a), SF-36 PCS score (c), SF-36 MCS score (e) and correlation between AQoL total score and SF-36 PCS score (g) and SF-36 MCS score (i) at baseline and correlation between changes from 0–4 months, 4–8 months and 8–12 months of EQ-5D index score and changes of AQoL total score (b), SF-36 PCS score (d), SF-36 MCS score (f) and changes of AQoL total score and SF-36 PCS score (h) and SF-36 MCS score (j) (all data points together); n = 84; Δ, change in.

Furthermore, at baseline, generic health status questionnaires were moderately to strongly correlated. The correlations between the changes were weak to moderate while the changes in AQoL total score were not correlated with the changes in SF-36 PCS score (Figures 2a to 2j).

### Agreement

Table 5 shows the agreement of the direction of changes in SGRQ total scores with the direction of changes in EQ-5D index score, AQoL total score, SF-36 PCS and SF-36 MCS scores. The direction of changes in SGRQ total scores agreed slightly (Kappa ranged from 0.12 to 0.18, p <0.05) with the direction of changes in EQ-5D index score, AQoL total score and SF-36 PCS score.

**Table 5:** Direction of 4-months changes in SGRQ total score and EQ-5D index score, AQoL total score, SF-36 PCS and SF-36 MCS

		**SGRQ total score**	**Improvement**	**Deterioration**
		**No change**		
change in EQ-5D	No change	12 (4.8%)	23 (9.1%)	24 (9.5%)
index score	Improvement	10 (4.0%)	52 (20.6%)	31 (12.3%)
**(К = 0.18)**	Deterioration	12 (4.8%)	32 (12.7%)	56 (22.2%)
change in AQoL	No change	5 (2.0%)	6 (2.4%)	14 (5.6%)
total score	Improvement	14 (5.6%)	54 (21.4%)	38 (15.1%)
**(К = 0.11)**	Deterioration	15 (6.0%)	47 (18.7%)	59 (23.4%)
change in	No change	7 (2.8%)	19 (7.5%)	12 (4.8%)
SF-36 PCS score	Improvement	10 (4.0%)	48 (19.0%)	38 (15.1%)
**(К = 0.12)**	Deterioration	17 (6.7%)	40 (15.9%)	61 (24.2%)
change in	No change	7 (2.8%)	9 (3.6%)	10 (4.0%)
SF-36 MCS score	Improvement	17 (6.7%)	49 (19.4%)	43 (17.1%)
(К = 0.08)	Deterioration	10 (4.0%)	49 (19.4%)	58 (23.0%)

*Abbreviations*: please see legend Table 2.

К, Cohen’s Kappa; bold printed numbers show significant (p ≤0.05) results.

The direction of changes in EQ-5D index score agreed fairly with the direction of changes in AQoL total score and SF-36 PCS score (Kappa ranged from 0.21 to 0.34, p <0.05). The direction of changes in AQoL total score agreed moderately with the direction of changes in SF-36 PCS score (К = 0.41, p <0.001). There was no agreement between the direction of changes in SF-36 PCS score and the direction of changes in EQ-5D index score (К = 0.17, p = 0.07) and AQoL total score (К = 0.15, p = 0.17).

## Discussion

### Key findings

The current study shows that the SGRQ total score and the EQ-5D index score, AQoL total score, and SF-36 PCS score were moderately to strongly correlated at four consecutive time points in patients with advanced COPD, while SGRQ total scores were weakly correlated with SF-36 MCS scores at four, eight and 12 months after baseline. The correlations between changes over time were weak or even absent. This is probably due to a poor or absent agreement in the direction of changes in the different health status questionnaires.

Previous cross-sectional studies found moderate to strong correlations between disease-specific and generic health status questionnaires in patients with COPD [19,27]. The current longitudinal study extends these findings and shows that SGRQ total scores were moderately to strongly correlated with the EQ-5D index score, AQoL total score, and SF-36 PCS score at four consecutive time points during one year follow-up. SGRQ total scores were weakly correlated with SF-36 MCS scores at four, eight, and 12 months. This can be explained by the fact that the SF-36 MCS focusses on mental aspects of health status, while the SGRQ total score includes several components of disease-specific health status. These findings are in line with Katsura et al. who reported even no correlation (r = 0.19) between the SGRQ total score and SF-36 MCS score in elderly patients with COPD [45].

Generic health status questionnaires were moderately to strongly correlated, while the correlations between the changes were weak to moderate. Pickard et al. found similar data in a cross-sectional study including inpatients and outpatients diagnosed with COPD concerning the EQ-5D index score and SF-36 PCS and MCS score [19]. EQ-5D index score showed the best correlation with AQoL total score, at baseline and between changes over time. Holland et al. compared the AQoL and EQ-5D in elderly patients admitted to an acute hospital and found a comparable correlation of the baseline scores and changes between these two questionnaires as in the present study [46]. Thus, even the generic health status questionnaires lack correlation. Researchers and clinicians should consider carefully which questionnaire is in line with their individual study objectives and study population. To the best of our knowledge, the AQoL has not been used as generic health status questionnaire in patients with COPD before. The current findings may suggest that the AQoL can be used to assess health status in outpatients with advanced COPD. However, future research is needed to support these results.

To the best of our knowledge, this is the first longitudinal study exploring the correlations (at four consecutive time points as well as the changes between these time points) between the SGRQ and the EQ-5D, AQoL, and SF-36 in patients with advanced COPD. Previously, improved health status (measured by SGRQ and SF-36) was found in patients with COPD who followed pulmonary rehabilitation or received home care [47-49]. Thus, it seemed reasonable to hypothesize that changes in disease-specific and generic health status questionnaires were somehow correlated. However, in the present study, the correlations between the changes in SGRQ total scores and in AQoL total scores as well as SF-36 MCS scores were absent. The changes in SGRQ total score and EQ-5D index score and SF-36 PCS scores were weakly correlated. These weak or absent correlations can be explained by the fact that the direction of changes in SGRQ total scores and EQ-5D index score, AQoL total scores and SF-36 PCS score just slightly agree. There was even no agreement on the direction of change in SGRQ total score and SF-36 MCS score. The agreement on the direction of change in disease-specific and generic health status was about 45%, while in 25 to 35% of the patients the observed four months changes were in opposite direction (Table 5). Ritva et al. compared the changes measured by SGRQ and 15D, a 15 dimensional generic health status questionnaire, among asthmatic patients and concluded that these instruments agreed on direction of change in health status in 64.8% of patients. In 15.8% of the cases the changes were in an opposite direction. In the remaining patients, health status changed according to one questionnaire [50]. However, Ritva et al. assessed changes in health status during three years [50], while we studied changes in health status during one year.

The current results support that healthcare professionals should use disease-specific as well as generic instruments to gain insight into the disease-specific and general impact of the disease and to get an in depth understanding of patients’ health status. Indeed, disease-specific questionnaires include relevant questions related to the patient’s disease and may generally be more sensitive to the disease and small changes in health status [17]. Generic health status questionnaires are intended for general use and are universally applicable: they tend to cover a broad variety of aspects (e.g. functional states, perceptions, social opportunities) [14,17]. Indeed, disease-specific instruments may be more responsive to change in clinical status or more sensitive in distinguishing patients with different disease severities while generic measures may be more likely to detect unexpected events which are probably not related to the disease and detect effects of diverse aspects on a disease [5]. The current study revealed that patients who experienced one or more exacerbations during 12 months reported a worse SGRQ total score at baseline compared to patients who had no exacerbation during 12 months while there were no significant differences according to the generic measurements. This may illustrate the disease-specific qualities of the SGRQ: the SGRQ may be more sensitive to detect differences in clinical status in patients with respiratory diseases. On the other hand, the AQoL showed changes over one year in total score and several domain scores (e.g., “independent living” or “illness”) while the SGRQ did not show changes over one year. This underlines the fact that generic and disease-specific instruments measure different aspects and cannot replace each other. Combining both types of questionnaires allows healthcare professionals to identify targets for interventions as well as to evaluate the effects of these interventions [13].

Recently, Pakhale et al. constructed a new questionnaire (McGill-COPD Quality of Life Questionnaire) by combining items of a disease-specific questionnaire with qualities of a generic questionnaire to measure health status in COPD patients [51]. Combining items of different measures can reduce patients’ load and increase their attention and participation. But it is still not clear which questionnaires or items in terms of psychometric properties (e.g. validity, reliability, sensitivity to change) are most suitable to use for combination. The McGill-COPD Quality of Life Questionnaire suggests possibilities. However, this questionnaire needs to be validated before it can be used in clinical practice or further research.

Interestingly, participants in the present study reported a better generic health status over time according to the AQoL illness domain, while the AQoL total score showed worse generic health status. This was also true for some EQ-5D domains and the EQ-5D VAS score (Table 3). These findings show that domain scores as well as total scores should be examined to achieve a correct understanding of patients’ health status. Furthermore, our results may suggest that the AQoL is more sensitive to detect changes than the SF-36 or SGRQ. However, the current study did not focus on sensitivity of health status questionnaires. A lack of previous studies using the AQoL as generic health status measure underlines the importance of further research in this field.

The present study has some methodological considerations that should be considered in interpreting the results. First, the study population consists of a convenience sample of patients with advanced COPD. Thus, it remains unknown whether these data are applicable for patients with GOLD stage I or II. Second, about 20% of the baseline study sample withdrew from the study during one year follow-up. These patients were more dependent in personal care and reported a worse baseline generic health status than patients who completed the study. It may be possible that changes in disease-specific and generic health status were different among patients who completed the study and patients who withdrew from the study. However, total scores of the disease-specific questionnaire SGRQ were comparable with findings from previous studies [6,19]. Third, the duration of the follow-up period is limited. However, longitudinal studies among elderly with advanced chronic diseases are scarce and recruitment and sustained participation is challenging in this population [52,53]. Fourth, ceiling effects and floor effects in longitudinal studies can limit the results because changes cannot be adequately detected. However, the proportion of patients reporting the highest possible score on the disease-specific and generic questionnaires ranged from 1.2-8.3% for all data points, while none of the patients reported the lowest possible score on any of the disease-specific and generic health status questionnaires on any of the data points. Because ceiling effects and floor effects are considered to be present if more than 15% of the subjects score the highest or lowest possible value, respectively [54], the influence of ceiling effects and floor effects seems to be limited in the current study. Fifth, the current findings need to be interpreted in the light of the number of comparisons that were made in the present study. Nonetheless, multiple findings in the same direction, rather than a single statistically significant result, suggest that these are not due to chance alone. Moreover, ‘Bonferoni adjustments are at best, unnecessary and, at worst, deleterious to sound statistical inference’ [55].

## Conclusion and implications

The current study suggests that health care professionals should use disease-specific as well as generic instruments to gain insight into the disease-specific and general impact of the disease. This is necessary to identify targets for interventions with the aim to improve disease-specific and generic health status and to evaluate the effects of interventions. Future research is required to compare disease-specific and generic health status questionnaires in a broader population. In addition, the possibilities and characteristics of a combined instrument measuring health status in patients with advanced COPD should be studied.

## Abbreviations

AQoL: Assessment of Quality of Life instrument; BMI: Body mass index; COPD: Chronic Obstructive Pulmonary Disease; EQ-5D: European Quality of life-5 Dimensions; FEV_1_: Forced expiratory volume in the first second; GOLD: Global initiative for chronic Obstructive Lung Disease; HADS: Hospital Anxiety and Depression Scale; LTOT: Long-term oxygen therapy; SF-36: Medical Outcomes Study 36-Item Short-Form Health Survey; SGRQ: Saint George’s Respiratory Questionnaire.

## Competing interests

The authors declare that they have no competing interests.

## Authors’ contributions

SW was responsible for analysis and interpretation of data and drafting the article. DJAJ and MAS were responsible for conception and design, acquisition of data, analysis and interpretation of data and drafting the article. JMGAS, FMEF and EFMW contributed to the study conception and design and interpretation of data. All authors read and approved the final manuscript.
